# High Mobility Group Box 1 (HMGB1): Molecular Signaling and Potential Therapeutic Strategies

**DOI:** 10.3390/cells13231946

**Published:** 2024-11-23

**Authors:** Sayantap Datta, Mohammad Atiqur Rahman, Saisudha Koka, Krishna M. Boini

**Affiliations:** 1 Department of Pharmacological and Pharmaceutical Sciences, College of Pharmacy, University of Houston, Houston, TX 77204, USA; 2 Department of Pharmaceutical Sciences, Irma Lerma College of Pharmacy, Texas A&M University, Kingsville, TX 78363, USA; sskoka@tamu.edu

**Keywords:** HMGB1, cardiovascular diseases, renal dysfunction, receptor for advanced glycation end products (RAGE)

## Abstract

High Mobility Group Box 1 (HMGB1) is a highly conserved non-histone chromatin-associated protein across species, primarily recognized for its regulatory impact on vital cellular processes, like autophagy, cell survival, and apoptosis. HMGB1 exhibits dual functionality based on its localization: both as a non-histone protein in the nucleus and as an inducer of inflammatory cytokines upon extracellular release. Pathophysiological insights reveal that HMGB1 plays a significant role in the onset and progression of a vast array of diseases, viz., atherosclerosis, kidney damage, cancer, and neurodegeneration. However, a clear mechanistic understanding of HMGB1 release, translocation, and associated signaling cascades in mediating such physiological dysfunctions remains obscure. This review presents a detailed outline of HMGB1 structure–function relationship and its regulatory role in disease onset and progression from a signaling perspective. This review also presents an insight into the status of HMGB1 druggability, potential limitations in understanding HMGB1 pathophysiology, and future perspective of studies that can be undertaken to address the existing scientific gap. Based on existing paradigm of various studies, HMGB1 is a critical regulator of inflammatory cascades and drives the onset and progression of a broad spectrum of dysfunctions. Studies focusing on HMGB1 druggability have enabled the development of biologics with potential clinical benefits. However, deeper understanding of post-translational modifications, redox states, translocation mechanisms, and mitochondrial interactions can potentially enable the development of better courses of therapy against HMGB1-mediated physiological dysfunctions.

## 1. Introduction

High mobility group (HMG) is a non-histone chromosome-binding protein, which is subdivided into HMGA, HMGB, and HMGN based on molecular weight, structural analogy, and DNA-binding properties [1,2]. Also known as amphoterin, HMGB1 is the most abundant non-histone nucleoprotein of the HMGB family [2]. It is expressed in eukaryotic cells and exhibits a remarkable 99% homology in protein sequence between humans and rodents [3,4,5,6,7]. Based on its nuclear expression and shuttling from the nucleus to the cytoplasm, HMGB1 exhibits dual functionality: both as a non-histone nucleoprotein and as an inflammatory cytokine inducer [8]. On the one hand, at the intracellular level, HMGB1 binds with H1 histone and regulates transcription, chromatin remodeling [9], DNA repair [10,11], DNA replication, and telomerase activity [12]. On the other hand, extracellular HMGB1 is primarily released by necrotic cells and binds with pattern recognition receptors (PRR), functioning as a damage-associated molecular pattern (DAMP) molecule [13]. Several studies have shown that at the cytoplasmic level, HMGB1 is localized in the mitochondria and lysosome [14,15], primarily through the regulation of heat shock protein beta-1 (HSPB1) in murine embryonic fibroblasts and cardiac cells [16,17]. Cytoplasmic HMGB1 modulates cell death processes, viz., apoptosis and autophagy. Cytoplasmic HMGB1 impedes cleaved caspase-3 accumulation and interacts with Beclin1 to maintain autophagy and prevent apoptosis [17,18]. In fact, intestinal epithelium expressed HMGB1 downregulates STAT3 activation and augments autophagic cascades for protection from infection [19].

Genome editing techniques have outlined the cell-specific functions of HMGB1 [20,21,22]. HMGB1 exhibits varying functionalities in different cell types or even in different subtypes of the same cell type [23,24,25]. Studies along these lines have shown that for hepatocytes, HMGB1 attributes to alcoholic liver injury [26], sepsis [25], hepatocellular carcinoma [27], and fibrosis [28]. In fact, HMGB1 loss-of-function has also been reported to bear close association with ischemia–reperfusion injury onset, owing to the rise in nuclear instability and susceptibility to cellular death [9].

Studies reported to date have provided a clear understanding of HMGB1 structure, functional basis, and association with different disease conditions. However, the mechanistic understanding of HMGB1 release, translocation, and correlated signaling pathways remains poorly understood. Gaining deeper insights into the intricate details of HMGB1-associated signaling mechanisms can potentially lead to a better understanding of HMGB1 druggability and the development of novel therapeutic strategies for minimizing risk factors and improving HMGB1-associated disease treatment in the long term.

## 2. HMGB1—Molecular Structure and Functional Correlation

Located on chromosome 13q12, HMGB1 gene includes four intronic sequences and five exonic sequences [29]. The human HMGB1 protein is a highly conserved nuclear protein with 215 amino acids [30]. From a functional perspective, HMGB1 is categorized into three regions, the A-box, B-box, and C-terminus. Both the A-box and B-box are composed of 80–90 amino acids with analogous amino acid repeats and non-specific DNA-binding sites [30]. The A-box exhibits an anti-HMGB1 effect, functioning as a natural HMGB1 antagonist [31], with sites for heparin binding and proteolytic cleavage [32]. The B-box possesses pro-inflammatory response, eliciting structural components with binding sites for the Toll-like receptor 4 (TLR4) and receptor for advanced glycation end products (RAGE), and experiences an antagonistic effect from the A-box [30,33].

**Figure 1:**
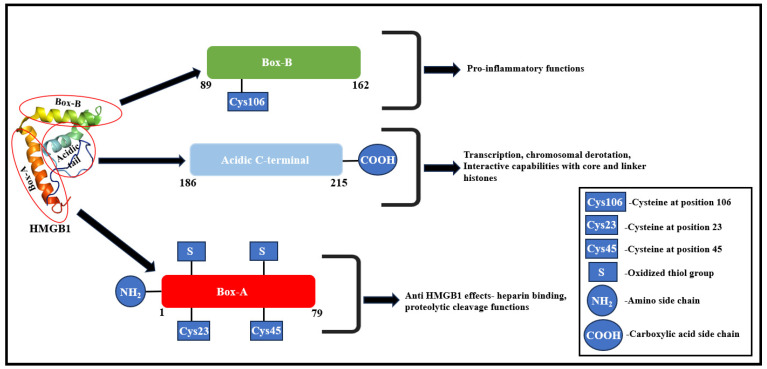
Molecular structure and functional correlation of HMGB1 domains. The Box-A chiefly exhibits anti-HMGB1 effects through specific intradomain regions, regulating heparin binding and proteolytic cleavage. The Box-B chiefly mediates pro-inflammatory functions. The acidic C-terminal regulates DNA-bending capabilities, chromosomal derotation, and the interactive potential of HMGB1 with core and linker histones [1,51,52,53].

A unique property of HMGB1 at the nuclear level is sequence-independent DNA binding and bending, which is accompanied by its ability to preferentially bind with distorted DNA [34,35]. DNA bending towards the major groove occurs because of minor groove binding and DNA intercalation of amino acid chains on the DNA-binding portions of the HMG box proteins [36]. The homogeneously acidic C-terminal decreases the DNA-binding affinity of the HMG box proteins [37,38]. This C-terminal is required for preferential binding to bent DNA and is necessary for governing cellular processes, viz., transcription and chromosomal derotation [39,40]. This acidic C-terminal tail also plays a crucial role in HMGB1 binding with chromatin via interactions with core and linker histones (Figure 1) [41]. In fact, its DNA-bending capabilities also enables HMGB1 to exhibit a regulatory chaperone function, facilitating DNA binding with other proteins [42,43].

Histone H1 and HMGB1 exhibit overlapping binding sites in chromatin and play a regulatory role in the overall chromatin organization [43]. Like H1, HMGB1 binds with linker DNA proximal to the nucleosome dyad but supports a looser chromatin organization when compared to H1 [44,45,46]. With two nuclear localization sequences (NLSs) and putative nuclear export signals (NESs), HMGB1 interacts with the nuclear receptor chromosome-region maintenance-1 (CRM-1), a nuclear transport receptor regulating the export and release of leucine-rich NES proteins from the nucleus to the cytoplasm [47]. Conserved lysine residues in the NLSs are susceptible to acetylation and can subsequently lead to nuclear exclusion and HMGB1 release [48,49]. Other PTMs regulating HMGB1 function include methylation, phosphorylation, glycosylation, ubiquitination, and Adenosine Diphosphate (ADP)-ribosylation [50].

## 3. Pathophysiology of HMGB1 Role in the Onset of Different Diseases

In view of its regulatory role in inflammatory cascades and immune responses, HMGB1 is often associated with the onset and/or progression of a wide variety of disease conditions

### 3.1. HMGB1 Role in Cardiovascular Complications

**Figure 2:**
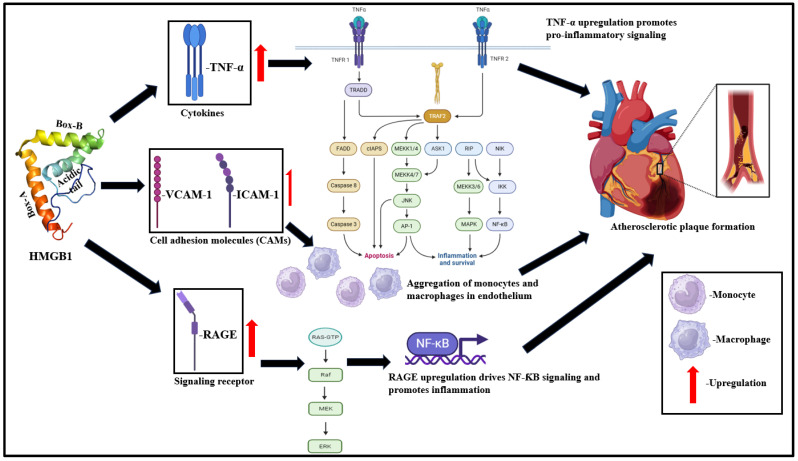
Schematic representation of HMGB1-induced signaling cascades culminating to atherosclerosis. Extracellularly released HMGB1 augments expression of cytokines (TNF-α), cell adhesion molecules (ICAM-1 and VCAM-1), and other signaling receptors (RAGE) to induce TNF-α pro-inflammatory signaling, monocyte, macrophage aggregation, NF-κB signaling. HMGB1-induced inflammation and concomitant decrease in anti-coagulant proteins like thrombomodulin lead to atherosclerotic plaque formation [51,53].

Inflammation induced by injury plays a pathologically important role in atherosclerosis initiation to plaque rupture and subsequent thrombotic complications [50,54]. Vascular damage initiates augmentation in the expression of endothelium-associated adhesion molecules like intercellular adhesion molecule (ICAM)-1 and vascular cell adhesion molecule (VCAM)-1 and drives aggregation of monocytes, macrophages, and platelets to the vessel wall [54,55]. HMGB1 is profusely expressed in vascular endothelial cells and is released passively from damaged endothelial cells [56]. Extracellularly released HMGB1 upregulates the expression of pro-inflammatory cytokines like tumor necrosis factor (TNF)-α, chemokines, viz., interleukin (IL)-8, monocyte chemoattractant protein (MCP)-1, and adhesion molecules, viz., ICAM-1 and VCAM-1, and RAGE [57,58,59]. ICAM-1 and VCAM-1 enable the aggregation of infiltrated macrophages and monocytes to the endothelium and, in turn, augments HMGB1 release [56,60]. Alongside the increase in HMGB1 levels, the expression of HMGB1-inhibiting proteins decrease rapidly, chiefly including anti-coagulant protein thrombomodulin [61]. This reduction in HMGB1-inhibiting protein levels play a crucial role in driving the onset of atherosclerosis (Figure 2) [51].

#### 3.1.1. Atherosclerosis

Atherosclerosis progression is usually driven by the transition of fatty streaks to focal thickening of the arterial inner layer [62]. This demands smooth muscle mobilization, and HMGB1 plays an important role in triggering chemotaxis of the smooth muscle [62]. Damaged endothelial cells lose their intrinsic anti-coagulant functions to enable platelet adherence to vascular endothelial cells [63]. Activated platelets release HMGB1 [63] and significantly contribute towards thrombus formation and subsequent atherosclerotic progression [64]. Macrophage aggregation, as discussed before, influence plaque rupture and the consequent formation of thrombus via plaque destabilizing factors like proteases [65]. Several studies also depict a potential upregulation in the expression of RAGE in macrophages of the atherosclerotic lesions with competitive inhibition of RAGE, attenuating atherosclerosis progression [66,67,68]. In summary, HMGB1 plays a crucial role not only in the onset of atherosclerosis but also drives its progression episodes.

#### 3.1.2. Adverse Left Ventricular Remodeling Post-Myocardial Infarction

Left ventricular (LV) remodeling is central to cardiac regeneration mechanisms post-myocardial infarction (MI) [69]. This is chiefly characterized by the restoration of cardiac structural homeostasis in response to myocardial ischemic injury and occurs in three distinct phases, inflammatory, proliferation, and maturation [70]. Underlining the overall complexity of LV remodeling, several studies show that HMGB1 is central to excessive post-MI inflammatory response, culminating in adverse LV remodeling and poor clinical outcomes [71,72]. The serum levels of HMGB1 and inflammatory cytokines, viz., TNF-α and IL-1β increase significantly in MI patients relative to chronic stable angina patients [73]. Several studies propose that active HMGB1 mRNA release occurs from the infiltrating immune cells, and this leads to extended post MI inflammatory signaling [74]. This also shows that HMGB1 mRNA remains in circulation for a prolonged period post-ischemic injury [74]. Excessive post-MI inflammatory response spreads to the non-infarcted area and contributes to adverse LV remodeling, accounting for heart failure, cardiomyocyte hypertrophy, apoptosis, and necrosis [75].

### 3.2. HMGB1 Role in Renal Dysfunction

Studies over the years outline a strong correlation between HMGB1 and pathogenesis of renal dysfunctions [7]. Circulating HMGB1 levels are elevated in acute kidney injury (AKI) patients and bears strong correlation with proteinuria and leukocyte count. In sepsis-associated AKI (SA-AKI) condition, HMGB1 plays a crucial role in driving sepsis via increase in glomerular fibrin deposition, renal tubular injury, and subsequent mortality [76,77,78]. From a signaling perspective, HMGB1 inhibits the activation of protein C pathway and augments coagulatory responses to distant organs in disseminated intravascular coagulation [DIC], a hallmark of septic conditions [79,80]. HMGB1 promotes the downregulation of bicarbonate absorption, especially in medullary thick ascending limbs [80]. This occurs primarily by influencing the RAGE-RhoA-ROCK1 signaling and inhibition of sodium/hydrogen exchanger-1 (NHE1) that culminates to the damage of the renal tubules [80]. HMGB1 also evokes a sepsis-associated inflammatory response of the renal tubular epithelial cells via the mitogen-activated protein kinase (MAPK) and nuclear factor kappa-light-chain-enhancer of activated B cells (NF-κB) signaling cascades, G1 cell cycle arrest, and increasing pro-inflammatory cytokine levels [81].

**Figure 3:**
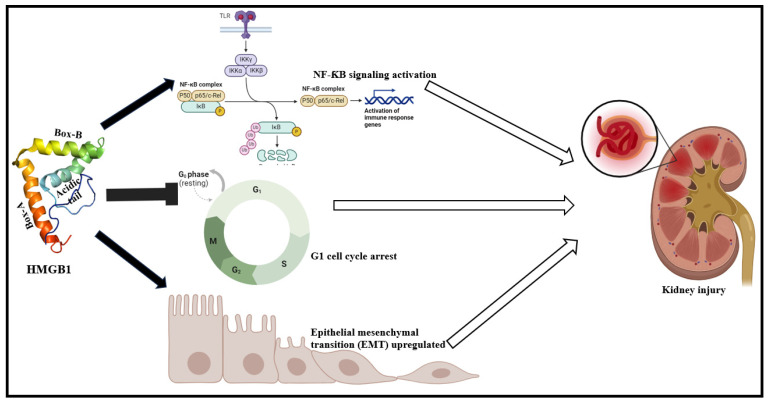
HMGB1-associated NF-κB signaling activation, G1 cell cycle arrest, and the augmentation of EMT (via RAGE signaling) culminates to kidney damage, attributing to subsequent renal dysfunctions [51,53].

Chronic kidney disease (CKD) also bears a strong association with HMGB1-elevated serum levels and decreases in estimated glomerular filtration rate (eGFR), being some of the hallmarks of CKD patients as well as in ambulatory peritoneal dialysis patients [82,83]. HMGB1 downregulates renal and vascular functioning and β-catenin silencing [84]. HMGB1 also promotes the epithelial–mesenchymal transition (EMT) phenotypic alterations of tubular cells via RAGE signaling (Figure 3) [85]. In the unilateral ureter obstruction (UUO)-induced renal fibrosis model, macrophages exhibit a rise in HMGB1 expression which accounted for a phenotypic switch to pro-inflammatory M1 subtype [86]. On the one hand, several studies also reveal that CCAAT/enhancer-binding protein (C/EBP) homologous protein (CHOP) modulates unilateral ureteral obstruction (UUO)-associated HMGB1 expression [87]. On the other hand, CHOP ablation has been reported to attenuate renal fibrosis via the downregulation of HMGB1/TLR4/ NF-κB pathway [87].

Several studies have also outlined a strong regulatory role of HMGB1 in driving the onset and progression of secondary renal damage, viz., glomerulonephritis, diabetic nephropathy (DN), and lupus nephritis (LN). Studies with crescentic glomerulonephritis mice model suggest that the rise in renal HMGB1 level occurs primarily via the nucleotide-binding oligomerization domain leucine rich repeat and pyrin domain containing 3 (NLRP3)/apoptosis-associated speck-like protein containing a caspase recruitment domain (ASC) inflammasome activation and local HMGB1 release in glomeruli [88,89]. Extracellularly secreted HMGB1 activates and drives NF-κB signaling [90]. This augments the release of pro-inflammatory cytokines like TNF-α, IL-6, and IL-1β in DN patients [91]. Serum examinations in such a scenario also reveal a rise in HMGB1 levels and correlated them with high IL-6 and IL-1β in serum [91]. HMGB1 also contributes to autoimmune renal dysfunction, which, upon release by dendritic cells (DCs), is primarily driven via the p38-MAPK signaling pathway [92]. Validation studies with microparticles (MP) reveal that MP-HMGB1 levels increase in blood, urine, and renal biopsy samples, underlining HMGB1 as a potential marker for patients with LN [93,94]. The proliferation of the mesangial cells, a key feature of LN, is also driven by HMGB1 via cyclin D1/CDK4/p16 and phosphoinositide 3-kinase (PI3K)/Akt signaling [95,96,97]. In fact, the TLR2/HMGB1 signaling axis also augments glomerular mesangial matrix deposition in LN through the MyD88/NF-κB signaling [98].

### 3.3. HMGB1 Role in Malignancies

As discussed before, HMGB1 promotes inflammatory injury and tissue damage. HMGB1 activates macrophages, monocytes, and neutrophils to increase the release of pro-inflammatory cytokines like IL-1, IL-6, and other macrophage inflammatory proteins in a p38- and MAPK-driven manner [99,100,101,102]. HMGB1 activation also upregulates the release of ICAM-1, VCAM-1, TNF-α, and IL-8, which corresponds to a rise in invasive and metastatic properties of cancer cells [57,58]. Studies reported to date have correlated HMGB1 upregulation with the onset and progression of melanoma, colon cancer, breast cancer, and pancreatic cancer, chiefly pointing towards the role of HMGB1 in augmenting neo-angiogenesis in cancerous tumor [103,104,105]. On the one hand, poor prognosis in prostate cancer patients have been correlated with HMGB1-driven RAGE signaling [106]. On the other hand, HMGB1 potentially augments the expression of transcription factors like extracellular signal-regulated kinase (ERK)-1/2 and p38 in lung cancer progression, regulating cancer cell proliferative capacity and tumor growth [107]. In driving other metastatic cancer progression, HMGB1 activates TLR4 and RAGE signaling, which in turn leads to caspase-1 activation that promotes cancer invasion and metastasis [108].

The ability of HMGB1 to bind and activate RAGE- and TLR4-associated pro-inflammatory signaling cascades induces cytokine upsurge, leukocyte recruitment, and consequently maintains the inflammatory bone marrow microenvironment [109]. This augments neoplastic transformation, supports tumor growth, invasion, and metastasis [110,111]. HMGB1 facilitates differentiation of myeloid-derived suppressor cells (MDSCs) in the bone marrow and impedes the protective activation of CD4^+^ and CD8^+^ T cells [112]. In fact, HMGB1 augments MDSC-associated IL-10 levels, crosstalk between MDSCs and macrophages, and downregulates T cell receptor L-selectin [112]. Extracellularly secreted HMGB1 increases the differentiation of pro-inflammatory M1 macrophages and interacts with circulating complements of C1q through RAGE and leukocyte-associated Ig-like receptor-1 (LAIR-1) [113]. Chronic lymphocytic leukemia (CLL) cells exhibit passive release of HMGB1 which are regulated through HMGB1/RAGE/TLR9 signaling and differentiate CD14^+^ monocytes to nurse-like cells (NLCs) [114]. Clinically, a high population of NLCs is associated with reduced survival of CLL patients. Increased HMGB1 secretion also correlates with cutaneous T cell lymphoma condition (CTCL), corresponding to increases in IL-4, IL-10, IL-19, and angiogenin [115]. In short, HMGB1 stimulation drives CTCL progression through angiogenesis and T-helper (Th)-2 polarization [115].

### 3.4. HMGB1 Role in Neurodegenerative Complications

HMGB1 exhibits a complicated spatiotemporal distribution pattern in the central nervous system (CNS). Both expression and release of HMGB1 is aggravated by varied courses of spinal cord and brain injuries [116]. Acute spinal cord injury models reveal increased HMGB1 levels in the cytoplasm of degenerating cells [117]. The expression profile of RAGE and TNF are also augmented by HMGB1, highlighting a mechanistic role of HMGB1 in inducing inflammation-based apoptotic upsurge [117]. HMGB1 is also one of the significant drivers of apoptotic degradation of motor neurons, as reported by studies on spinal cord ischemia model [118,119]. Such studies also outline the better survival of spinal cord motor neurons with reduction in serum HMGB1 levels [120,121]. Under chronic neurodegenerative processes, HMGB1 interacts with Mac1, activates microglia, and subsequently drives neuronal damage cascades, prominently highlighting HMGB1 as a target for treating acute and chronic CNS damages [122].

**Figure 4:**
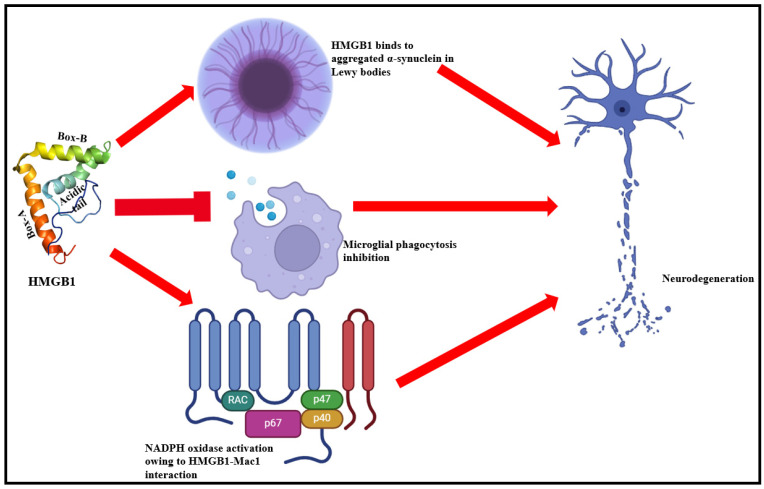
HMGB1 binds to α-synuclein aggregates in Lewy bodies, inhibits microglial phagocytosis, and upregulates NADPH oxidase levels (chiefly via NF-κB signaling) to mediate neurodegeneration [51,53].

HMGB1 has also been reported as a risk factor for other neurodegenerative conditions. It inhibits microglial phagocytosis and stabilizes Aβ42 oligomers in Alzheimer’s disease [123,124]. For memory impairment processes, HMGB1 chiefly triggers TLR4 and RAGE signaling [124]. In Parkinson’s disease, HMGB1 binds to aggregated α-synuclein in Lewy bodies and play a crucial role in augmenting chronic neurodegeneration (Figure 4) [125]. This occurs primarily via HMGB1 binding with Mac1, a microglial membrane receptor which, in turn, activates NF-κB signaling and expression of reduced nicotinamide adenine dinucleotide phosphate (NADPH) oxidase [122].

Increased HMGB1 expression has also been reported in the white mater of multiple sclerosis patients and in mice model populations of myelin-oligodendrocyte-induced autoimmune encephalomyelitis [126]. HMGB1 and its receptors, viz., TLR2, TLR4, and RAGE augment inflammatory responses and promote neuroinflammation cascades in demyelination [127].

### 3.5. HMGB1 Role in Autoimmune Diseases

#### 3.5.1. Rheumatoid Arthritis (RA)

HMGB1 exhibits abnormal extranuclear expression in RA patients’ serum, synovial fluid, and associated synovial tissues [128,129,130]. This abnormal expression profile is typical in the vascular endothelial cells and macrophages of RA patients [131]. Concomitantly, synovial fluid macrophages show augmented expression of TLR2, TLR4, and RAGE [132]. In fact, HMGB1-induced RAGE activation releases TNF, IL-1β, and IL-6 [132]. Functional studies of HMGB1 and IL-1β reveal evidence for HMGB1-induced transactivation of IL-1β promoter [133]. This promoter has two transcription factor binding sites, one for nuclear factor IL-6 and the other for PU.1, which is a myeloid and B cell specific transcription factor belonging to the Ets family [134]. HMGB1 interacts with PU.1 and subsequently results in the activation of IL-1β. Additionally, HMGB1 binds with bacterial DNA, viral RNA, endotoxin, and other microbial molecules, and the resultant complex elicits a pro-inflammatory cytokine upsurge. This also initiates the onset of chronic arthritis [134].

In collagen type II-induced arthritis (CIA), HMGB1 has been reported to be significantly augmented in the extracellular space and cytoplasm of macrophages, synoviocytes, fibroblasts, and vascular endothelial cells [135]. High HMGB1 levels in pannus are primarily caused by tissue ischemia, activated complement protein, and by pro-inflammatory cytokines such as TNF and IL-1 [128]. Such high levels of HMGB1 in pannus tissues contributes to the onset and progression of destructive cartilage and bone penetration by facilitating the activities of tissue plasminogen activator and metalloproteinases [136,137].

#### 3.5.2. Systemic Lupus Erythematosus (SLE)

SLE chiefly involves autoantibody production and multiple-organs-based systemic inflammation. Several studies reveal that HMGB1 is significantly upregulated in blood samples collected from SLE patients [138,139]. Upregulated HMGB1, in the cytoplasmic and extracellular spaces of the skin lesions in SLE patients, also correlates with elevated levels of IL-1β and TNF [140,141]. Ultraviolet radiation, in fact, has been reported to induce HMGB1 translocation into the cytoplasmic domain and drive SLE onset [142]. The HMGB1–DNA complexation induces anti-DNA antibody production under DNA inactivation, chiefly via the TLR2 signaling cascade [143]. HMGB1 also induces augmented expression of TLR2 and TLR4 in the peripheral blood mononuclear cells of SLE patients [144,145]. The rise in TLR4 and TLR7 levels has been correlated with autoantibody production and the onset of LN [146]. TLR7, in fact, bears close association with the upregulation of IFN-α and IFN-β expression in systemic lupus erythematosus (SLE) patients [147]. On the one hand, TLR8 upregulation also accounts for the onset of SLE glomerulonephritis [148]. On the other hand, activation of TLR9 is a potential therapeutic strategy against SLE with recent studies highlighting TLR9 deletion culminating to the augmentation of lymphocyte and plasmacytoid DC, which is accompanied by elevation of IgG and IFN-α serum [149]. Several studies also reveal that point mutation of the TLR9 gene (TLR9^P915H^, lacking MyD88) culminate to a more prominent decline in SLE disease progression when compared to wild-type populations [150]. Such findings outline a pro-inflammatory effect of TLR9-MyD88 signaling and the selective disruption of this signaling delays the onset of SLE [150].

#### 3.5.3. Type 1 Diabetes Mellitus (T1DM)

Progressively damaged pancreatic β-cells, a characteristic of T1DM and mediated chiefly by immune cells, exhibit passive release of HMGB1 [151]. During autoimmune responses, HMGB1 can also be actively secreted by immune cells infiltrating the islet cells [151]. Several studies show that HMGB1 serum levels are augmented in both mice model and patients with T1DM [151,152,153]. From a signaling point of view, HMGB1 potentially augments autoimmune responses through TLR4 upregulation and the destabilization of regulatory T cell functionality [154]. Extracellularly increased HMGB1 in T1DM patients also accounts for the increased expression of TLR2 and TLR4 on the surface of peripheral blood mononuclear cells [155]. Investigative studies in this regard show higher expression of TLRs, MyD88, toll/interleukin-1 receptor domain-containing adaptor inducing interferon-β (TRIF), and other downstream signaling proteins in T1DM patients which included the secretory levels of IL-1 and TNF-α [156].

### 3.6. Metabolic Syndrome (MetS)

Over the past few decades, the global prevalence of metabolic syndrome (MetS) has exhibited an alarming rise in cases, chiefly because of the global increase in childhood obesity and diabetes [157,158]. Several studies aimed at identifying potential biomarkers outlining the prodromal phase of MetS development show that HMGB1, an autophagy regulator, plays a significant role towards the onset of MetS [159,160]. As an autophagy regulator, HMGB1 modulates the expression of heat shock protein β-1, competes with BCl-2 for interaction with Beclin-1, and promotes Beclin-1-mediated autophagosome assembly [16,161]. In adipocytes, c-Jun regulates HMGB1 secretion which bears strong correlation with non-alcoholic fatty liver disease (NAFLD) onset, primarily driven by the downstream effectors of insulin receptor [162,163,164]. Multiple studies propose a linear association between HMGB1 levels and MetS onset, accounting for the increased NF-κB and homeostasis model assessment of insulin resistance (HOMA-IR) activity [165,166,167]. Significantly increased HMGB1 levels have been observed in obese children and correlates with increased IL-6, TNF-α, IL-18, and adiponectin levels, which are potentially driven via RAGE activation [168]. However, a clear understanding of how HMGB1 associates with MetS and associated complications remains poorly understood.

## 4. Therapeutic Perspectives

### 4.1. Anti-HMGB1 Antibodies

Anti-HMGB1 polyclonal and monoclonal antibodies are utilized to antagonize extracellularly released HMGB1, especially in sepsis [169,170]. Polyclonal anti-HMGB1 antibodies have also been implicated in confronting against inflammatory upsurge, arthritis, acute pancreatitis, and inflammatory bowel disease [171,172,173]. Studies with experimental models of hemorrhagic shock reveal that polyclonal anti-HMGB1 antibodies prolonged survival and hindered the onset and progression of gut barrier dysfunction [174]. Polyclonal anti-HMGB1 antibodies have also been reported to decrease lung inflammation and fibrosis in bleomycin-induced pulmonary fibrosis study models [175]. The administration of polyclonal anti-HMGB1 antibodies also attenuates liver injury, promotes liver regeneration cascades, and supports the hepatic immune profile in an experimental murine model of hepatotoxicity [176]. Polyclonal anti-HMGB1 antibodies have also been implicated in neuropathic pain relief, reduction in neuronal degeneration, downregulation of pro-inflammatory TNF-α, and the restoration of glycemic control in diabetes [120,177,178].

**Figure 5:**
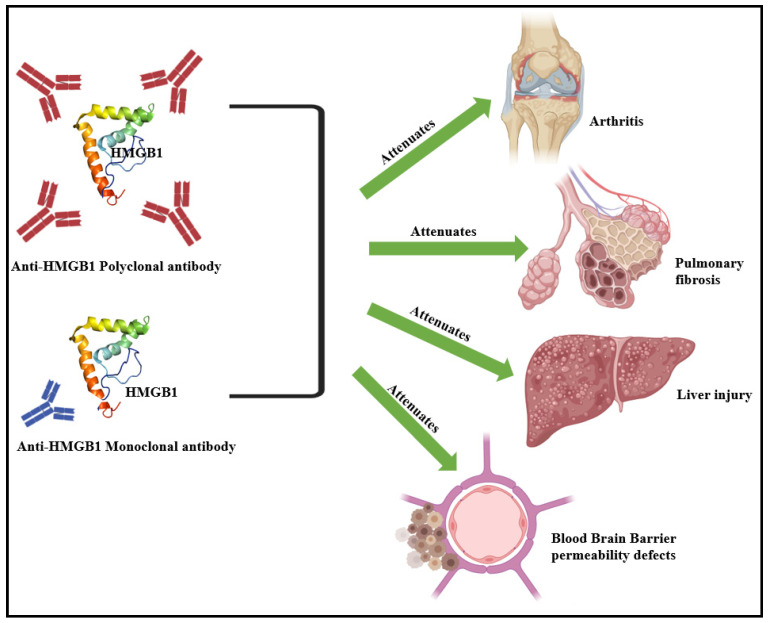
Polyclonal- and monoclonal-antibody-mediated HMGB1 targeting attenuates the onset and progression of varied dysfunctions, viz., arthritis, drug-induced pulmonary fibrosis, hepatic injury, and BBB defects [51,53].

Considering the disadvantages associated with polyclonal antibody usage, viz., increased cross-reactivity, non-specific antigen reactions [179], and immunological status of the host [179], monoclonal anti-HMGB1 antibodies have been employed over the years for improving therapeutic outcomes [180,181]. The monoclonal antibody (mAb) m2G7, targeting amino acids 53 and 63 in the A-box domain of HMGB1, has been implicated for sepsis [182], pancreatic islet graft transplantation, arthritis, and drug-induced liver injury [183,184]. Anti-HMGB1 mAbs, which precisely targets the HMGB1 C-terminal domain, have also been developed [185]. These have been utilized against brain infarction and reduced blood–brain barrier (BBB) permeability conditions (Figure 5) [186]. The same anti-HMGB1 mAbs have also elicit neuroprotective functions in treating neuropathic pain and Parkinson’s disease [187]. Anti-HMGB1 mAbs have also been developed to target the 17-mer peptide P1 domain in the B-box domain of HMGB1 (DPH1.1 mAb). These antibodies have been reported to restrict HMGB1-associated 3T3 fibroblast migration and leukocyte recruitment [188,189]. This, in turn, minimizes risks associated with the onset and progression of hepatitis B [190]. The DPH1.1 mAb also attenuates diet-induced atherosclerosis under apolipoprotein (Apo) E deficiency and elicits protection against blood–brain barrier (BBB) damage, associated brain edema, and against mesothelioma [191,192,193].

### 4.2. Soluble RAGE (sRAGE)

As discussed before, extracellularly released HMGB1 strongly influences RAGE signaling by inducing HMGB1-associated physiological dysfunctions [194]. Biochemical studies reveal that RAGE has multiple isoforms, viz., full-length RAGE, N-terminal truncated RAGE, and C-terminal truncated RAGE [195]. These isoforms arise primarily due to alternative splicing [195]. Of these, the C-terminal truncated RAGE isoform is released extracellularly but lacks the transmembrane domain, thereby being referred to as soluble RAGE (sRAGE). This property of sRAGE is clinically exploited to serve as an inveigle receptor for HMGB1 binding [196]. It blocks HMGB1/RAGE signaling and reduces inflammation as reported in studies employing diabetes-induced atherosclerosis [68]. sRAGE also promotes hepatic regeneration [197] and restricts bacterial translocation to lymph nodes during hemorrhagic shock and downregulates IL-6 [198]. Several studies also outline the role of sRAGE in attenuating diabetes-associated microvascular and macrovascular dysfunctions [199,200,201].

### 4.3. Peptides and Peptidomimetics

Peptides and peptidomimetic biologics constitute a novel and improved course of therapy against HMGB1-associated dysfunctions. Studies in this context have successfully developed peptides (P5779) that mediates the selective inhibition of HMGB1 interaction with extracellular TLR4 adaptor and myeloid differentiation factor (MD-2) without non-specifically affecting other TLR4/MD-2 ligands [202]. The targeted epitope has been identified to be cysteine 106 in the B-box of HMGB1, which is otherwise important for HMGB1-associated cytokine upsurge [203]. Murine model studies reveal that peptide-based HMGB1 targeting alleviated multiple dysfunctions, viz., attenuating the upsurge of alanine aminotransferase (ALT), aspartate aminotransferase (AST) [202], pro-inflammatory cytokines [204], and progression of liver necrosis [205]. Peptide based therapy has also been implicated in sepsis and murine models of intimal hyperplasia [204].

### 4.4. Small Molecule Inhibitors (SMIs)

Over the years, small molecule inhibitors (SMIs) have been developed to target HMGB1-associated pro-inflammatory signaling cascades. SMI-based targeting chiefly focuses either on HMGB1-specific binding or attenuating the extracellular release of HMGB1.

#### 4.4.1. SMIs Against Extracellular HMGB1 Release

The most notable SMI developed against HMGB1 extracellular release is ethyl pyruvate, an aliphatic ester developed from pyruvic acid [206,207]. Besides targeting the extracellular release of HMGB1, ethyl pyruvate also attenuates nuclear–cytoplasmic translocation of HMGB1 [173,208]. Multiple studies have also outlined potential therapeutic roles of HMGB1 in infection and injury [209]. Ethacrynic acid, a diuretic, has been reported to intervene with HMGB1-associated inflammasome activation [210,211]. Another SMI, inflachromene (ICM), interferes with cytoplasmic localization and extracellular release of HMGB1 by altering the PTMs of HMGB1 [212,213,214]. In doing so, ICM prevents pro-inflammatory signaling induced by HMGB1 and attenuates microglia-associated neuronal damage [215,216].

Naturally obtained SMIs targeting HMGB1 release have also proved to be useful in attenuating HMGB1-induced lethal systemic inflammation [217]. These include (-)-epigallocatechin-3-gallate (EGCG), quercetin, and lycopene. EGCG has been reported to rescue the effects of HMGB1-induced endotoxemia and sepsis by preventing systemic accumulation of HMGB1 and its release on macrophage surface [218]. Quercetin attenuates both the release and subsequent cytokine activation of HMGB1 [219]. Lycopene has been reported to downregulate lipopolysaccharide (LPS)-mediated release of HMGB1, HMGB1-associated TNF secreting phospholipase A2, and HMGB1-induced pro-inflammatory cascades [220]. Mechanistically, lycopene mediates these through the inhibition of cell adhesion molecules (CAMs), TLR-2, TLR-4, and RAGE [220].

**Figure 6:**
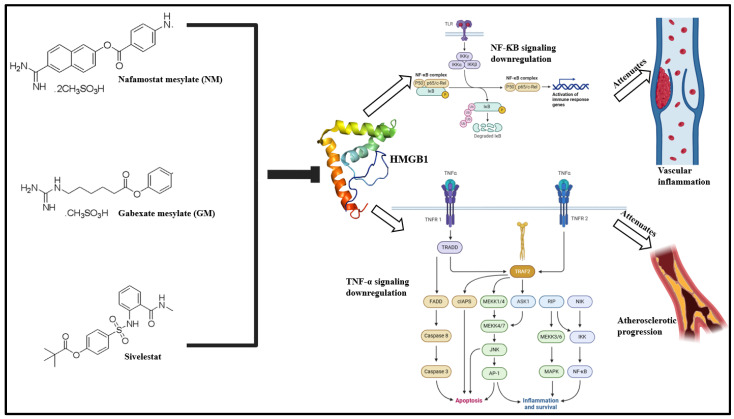
Synthetically derived SMIs, viz., nafamostat mesylate, gabexate mesylate, and silvestat prevent extracellular HMGB1 release, downregulate NF-κB and TNF-α pro-inflammatory signaling, and attenuate vascular inflammation and atherosclerosis progression [51,53,225].

Synthetically derived SMIs, viz., nafamostat mesylate (NM), gabexate mesylate (GM) and sivelestat sodium hydrate have been reported to attenuate LPS-associated HMGB1 release [217,221]. This results in the inhibition of cytokine release and inhibition of NF-κB signaling [222]. SMIs of the statin group, atorvastatin and simvastatin, have also been reported to downregulate HMGB1/RAGE signaling and attenuate vascular inflammation, atherosclerotic progression via decrease in HMGB1 expression, and release (Figure 6) [223,224].

#### 4.4.2. SMIs Targeting HMGB1 Binding

Naturally obtained glycyrrhizin (Gly), a triterpenoid produced by the licorice plant *Glycyrrhiza glabra*, inhibits the phosphorylation and DNA binding of HMGB1 [226]. It has been reported to bind to the concave surface at the junction of the arms of the HMGB boxes proximal to the DNA binding site [227,228]. This binding mainly affects the positively charged Arg24 and Gln20 residues, which is mediated by covalent interactions with the carboxylic skeleton of Gly and the Van der Waals forces between HMGB1 hydrophobic residues and the triterpene core of Gly [227]. Several studies also reveal that prior treatment with Gly attenuates trimethylamine N-oxide (TMAO)-induced HMGB1 and TLR4 expression [229]. Gly has been reported to exhibit protective functions in the brain and hepatic inflammatory progression [230,231]. Carbenoxolone, an aglycone derivative of Gly and another reported HMGB1 binder, has been utilized for ameliorating inflammation, peptic, and esophageal ulcerations [232,233]. In fact, the core structural skeleton of Gly has also been exploited to design and develop cardioprotective agents [234,235]. The cardioprotective functions have been characterized to be mediated chiefly via HMGB1 binding and the downregulation of HMGB1 expression [236,237].

Salicylic acid (SA), a well-established anti-inflammatory drug, has been reported to bind with the A-box and B-Box domains of HMGB1 [238]. Several studies confirm such binding with targeted mutations in HMGB1, inhibiting SA binding with HMGB1 and turning HMGB1-induced cellular migration resistant to SA [238]. SA derivatives have also been reported to exhibit HMGB1 binding and subsequently downregulate HMGB1-associated cytokine induction. From a genetic perspective, HMGB1 has been found to induce the expression of *Ptgs2* gene, which is otherwise responsible for encoding the cyclooxygenase (COX)-2 protein [238,239,240]. SA not only binds with HMGB1 but also downregulates COX-2 induction in attenuating inflammation. Beyond COX-2 downregulation, the addition of hydrophobic moieties to SA augments HMGB1 inhibition [241]. This has been implicated in the SA-associated attenuation of malignant mesothelioma (MM), whereby HMGB1 has been identified as a critical regulator in the onset and progression of MM [242].

## 5. Current Limitations

Extensive investigations have shown that HMGB1 plays a critical role in infection and injury associated with the onset of inflammation. Clinically, HMGB1 serum serves as an important biomarker for sepsis and associated organ dysfunction onset [170]. Studies chiefly investigating signaling crosstalk enables considerable understanding of HMGB1-associated signaling cascade in the onset of inflammatory and progression [71,243,244,245]. However, existing scientific gap limits HMGB1 druggability and its potential application clinically. The exact mechanism governing nucleus/cytoplasm shuttling of HMGB1 and eventually into the extracellular matrix remains poorly understood [246]. The different redox states of HMGB1 makes it potentially vulnerable to in situ modifications, challenging clear understanding of the exact functions of HMGB1 isoforms [247,248]. As discussed before, HMGB1 and its isoforms exhibit cell type specificity, complicating the understanding of the exact role of HMGB1 inhibition in varying disease conditions [249,250]. Functional analysis has enabled understanding of the role of active and passive modes of extracellular HMGB1 release [251,252]. However, no mechanistic understanding to distinguish between the two modes of HMGB1 release exists so far. This makes it difficult to understand which of these needs to be targeted for better therapeutic outcome [243,253,254,255]. To comprehend this from an immunological perspective, functional difference in immune cell versus non-immune cell released HMGB1, potential consequences of manipulating translocation versus release of HMGB1, and impact of HMGB1-associated immune responses remains less understood [52,256]. Lysosomal localization and associated structural understanding for HMGB1 also remains obscure, with better understanding promising to yield potentially useful clinical directions for anti-HMGB1 based therapy [246,256].

From a mitochondrial perspective, HMGB1 mediates mitochondrial fission via the activation of ERK-1/2 signaling, culminating to pulmonary arterial hypertension (PAH) onset and progression [257]. In fact, mesenchymal stem cells (MSCs) exhibit protection of T cell acute lymphoblastic leukemia (T-ALL) cells from drug-induced apoptosis. This occurs primarily via mitochondrial transfer from Jurkat cells to MSCs, culminating to chemotherapeutic resistance [258]. Although mechanistic studies reveal that such mitochondrial transfer is mediated by intercellular tunneling nanotubes (TNTs) between the Jurkat cells and MSCs, whether such mechanism guides other HMGB1-associated dysfunctions remains poorly known [258,259]. Extracellularly released HMGB1 has been revealed to translocate into elongated mitochondria and mediate status epilepticus (SE)-induced CA1 neuronal damage [14,260,261,262]. Although reactive oxygen species (ROS)- and reactive nitrogen species (RNS)-induced oxidative and nitrosative stress serve as major pathophysiological drivers for HMGB1-associated dysfunction, the role of superoxide dismutase (SOD) mimicking drug candidates remains vastly unexplored [263,264]. SOD mimicking agents can potentially confront ROS and RNS and outline potentially useful protective mechanisms to attenuate inflammation-associated cell death owing to HMGB1 inhibition.

## 6. Future Perspectives

Studies aimed at understanding the redox modifications of HMGB1 and its correlation with HMGB1 pathophysiology constitute a major direction for future studies. Studies focusing on the spatiotemporal distribution patterns of HMGB1 can also be undertaken to clearly understand the mechanism(s) governing the nucleus/cytoplasm shuttling of HMGB1. Recent studies reveal that HMGB1 and TLRs are strongly associated with inflammasomes, pyroptosis, and ferroptosis [240,265,266]. Future courses of research can potentially explore the mechanism(s) that govern HMGB1 entry into the cells and subsequent intracellular binding with TLRs. Such studies can potentially explain HMGB1 release patterns and enable the understanding of functional differences between active and passively released HMGB1. At the mitochondrial level, studies beyond developing SOD mimics can aim towards explaining the exact mechanism by which mitochondrial peroxide levels drive HMGB1 secretion and release, potentially identifying novel druggable targets [267].

## 7. Conclusions

In summary, the current paradigm of studies reveal that HMGB1 is a major driver in inflammation-associated physiological dysfunctions, primarily interacting with TLRs and RAGE. Biologics, both naturally obtained and synthetically derived, have targeted HMGB1 in ameliorating a broad spectrum of physiological dysfunctions. However, clarity regarding the exact mode of extracellular HMGB1 release and mitochondrial perspectives involved in HMGB1 pathophysiology remains largely obscure. Future courses of studies addressing these scientific gaps can potentially elucidate druggable HMGB1 signaling cascades and provide deeper clinical insights into HMGB1-targeted therapy.

## Data Availability

No new data were created or analyzed in this study.
